# An age-related numerical and functional deficit in CD19^+^CD24^hi^CD38^hi^ B cells is associated with an increase in systemic autoimmunity

**DOI:** 10.1111/acel.12114

**Published:** 2013-07-19

**Authors:** Niharika A Duggal, Jane Upton, Anna C Phillips, Elizabeth Sapey, Janet M Lord

**Affiliations:** 1 MRC-ARUK Centre for Musculoskeletal Ageing Research, School of Immunity and Infection, Birmingham University Medical SchoolBirmingham, B15 2TT, UK; 2 School of Sport and Exercise Sciences, Birmingham University Medical SchoolBirmingham, B15 2TT, UK; 3 School of Clinical and Experimental Medicine, Birmingham University Medical SchoolBirmingham, B15 2TT, UK

**Keywords:** autoimmunity, B cells, cellular immunology, inflammation, rheumatoid factor

## Abstract

Autoimmunity increases with aging indicative of reduced immune tolerance, but the mechanisms involved are poorly defined. In recent years, subsets of B cells with immunoregulatory properties have been identified in murine models of autoimmune disorders, and these cells downregulate immune responses via secretion of IL10. In humans, immature transitional B cells with a CD19^+^CD24^hi^CD38^hi^ phenotype have been reported to regulate immune responses via IL10 production. We found the frequency and numbers of CD19^+^CD24^hi^CD38^hi^ cells were reduced in the PBMC pool with age. IL10 expression and secretion following activation via either CD40, or Toll-like receptors was also impaired in CD19^+^CD24^hi^CD38^hi^ B cells from healthy older donors. When investigating the mechanisms involved, we found that CD19^+^CD24^hi^CD38^hi^ B-cell function was compromised by age-related effects on both T cells and B cells: specifically, CD40 ligand expression was lower in CD4 T cells from older donors following CD3 stimulation, and signalling through CD40 was impaired in CD19^+^CD24^hi^CD38^hi^ B cells from elders as evidenced by reduced phosphorylation (Y705) and activation of STAT3. However, there was no age-associated change in expression of costimulatory molecules CD80 and CD86 on CD19^+^CD24^hi^CD38^hi^ cells, suggesting IL10-dependent immune suppression is impaired, but contact-dependent suppressive capacity is intact with age. Finally, we found a negative correlation between CD19^+^CD24^hi^CD38^hi^ B-cell IL10 production and autoantibody (Rheumatoid factor) levels in older adults. We therefore propose that an age-related decline in CD19^+^CD24^hi^CD38^hi^ B cell number and function may contribute towards the increased autoimmunity and reduced immune tolerance seen with aging.

## Introduction

B cells have been shown to play a pathogenic role in human autoimmune diseases due to their ability to secrete autoantibodies and serve as antigen-presenting cells to T cells (Yanaba *et al*., 2008b). Recently, evidence has emerged for a regulatory role of B cells in limiting immune responses, analogous to the suppressive activity of regulatory T cells. Janeway and colleagues suggested that B cells were involved in suppression of inflammation, reporting that the absence of B cells exacerbated the course of T-cell-mediated autoimmune reactions (Wolf *et al*., 1996). Thus, B cells can play both protective and pathogenic roles in the same pathological setting. Since then a subset of B cells with immunosuppressive properties have been isolated and shown to suppress inflammation in a number of murine models of chronic inflammation, including collagen-induced arthritis (CIA), experimental autoimmune encephalitis (EAE) and inflammatory bowel disease (Mauri & Bosma, 2012). Two phenotypically distinct subsets of B cells: transitional CD19^+^CD24^hi^CD38^hi^ B cells (Blair *et al*., 2010) and CD19^+^CD5^+^CD1d^hi^ ‘B10’ B cells (Yanaba *et al*., 2008a) have been demonstrated to exert immunosuppressive functions. Recently, an additional B-cell subset of CD24^hi^ CD27^+^ B cells, with 60% of these cells expressing CD38, has been identified with characteristics similar to mouse B10 cells (Iwata *et al*., 2011).

In humans, CD19^+^CD24^hi^CD38^hi^ and CD19^+^CD5^+^CD1d^hi^ B cells mediate their immunosuppressive effects via secretion of the regulatory cytokine IL10 and/or by contact-dependent interaction with pathogenic T cells (Mizoguchi & Bhan, 2006). IL10 plays an essential role in maintaining immune homoeostasis. Its biological functions include downregulation of antigen presentation by macrophages and DCs and suppressing production of pro-inflammatory cytokines by CD4 T cells, monocytes and macrophages (Moore *et al*., 2001). IL10-producing CD19^+^CD24^hi^CD38^hi^ B cells are also able to promote expansion of IL10-producing Foxp3^+^ regulatory T cells and have been reported to play a role in inducing recruitment of T_reg_ cells to the site of inflammation (Mann *et al*., 2007). Human IL10-producing B cells have also been shown to regulate innate immune responses by reducing TNFα production by monocytes (Iwata *et al*., 2011).

The B7 family of costimulatory molecules B7-1(CD80) and B7-2 (CD86) is capable of regulating T-cell growth, survival and differentiation via ligation to CD28 and cytotoxic T lymphocyte protein 4 (CTLA4) receptor on T cells (Collins *et al*., 2005). In addition to IL10 production, the suppressive effect of CD19^+^CD24^hi^CD38^hi^ B cells is also mediated via engagement of CD80 and CD86 (Blair *et al*., 2010; Mauri & Bosma, 2012). For example, B7 expression by B cells is required for mediating recovery in murine models of autoimmunity by mediating peak expression of IL10 and Foxp3 (Salomon & Bluestone, 2001). Additional IL10-independent protective functions in B-cell subsets include expression of granzyme B by a subset of CD5^+ve^ B cells, expression of FasL after activation by mitogens, and a subset of TGF secreting B cells have also been identified (Klinker & Lundy, 2012). Interestingly, *Salmonella* infection also results in rapid differentiation of IL10 expressing plasma-cell-like B cells (CD19^+^ CD138^+^), involving TLR signalling (Neves *et al*., 2010).

In this study, we have focussed upon the CD19^+^CD24^hi^CD38^hi^ and CD19^+^CD5^+^CD1d^hi^ B cells due to their established role in autoimmune pathogenesis in rodent models and their reduced activity in autoimmune disease in humans (Blair *et al*., 2010; Iwata *et al*., 2011). Development, maturation and expansion of B cells with immunosuppressive properties require specific external signals (Mauri & Bosma, 2012). CD40 engagement plays a pivotal role in their generation and function, halting B-cell differentiation into plasma cells and facilitating differentiation into cells with regulatory properties (Randall *et al*., 1998). In addition to T-cell-dependent stimulation, B cells can also be activated by microbial products via Toll-like receptor (TLR) signalling (Yanaba *et al*., 2009). Expression of TLR1, TLR2, TLR4, TLR7 and TLR9 transcripts has been reported on B cells (Barr *et al*., 2007), and TLR-activated B cells are capable of resolving autoimmune disorders by inhibiting pathogenic T cells via IL10 production (Lampropoulou *et al*., 2008).

The incidence of autoimmune conditions such as rheumatoid arthritis (RA) increases with age and is also characterized by pathological features typical of age-related immune dysfunction, termed immunesenescence (Lindstrom & Robinson, 2010). This raises the possibility that immunesenescence may be a contributing factor in the development of autoimmunity. Age-related T-cell senescence has been associated with autoimmunity, and an inverse relationship has been reported between thymic capacity and the incidence of RA (Weyand *et al*., 2003). Naturally occurring CD4^+^CD25^+^Foxp3^+^ regulatory T cells play an essential role in regulating immune responses and preventing autoimmunity. Although older adults are characterized by higher numbers of T_regs_, a decline in the per cell immune suppressive activity of these cells has been reported (Dejaco *et al*., 2006). Reduced immune tolerance with age is also supported by reports of higher autoantibody levels such as rheumatoid factor, in healthy older adults (Moulias *et al*., 1984). It has also been hypothesized that self-reactive memory B cells accumulated with age may become reactivated later in life due to deficiency in immune tolerance leading to elevated autoantibody levels in the elderly (Stacy *et al*., 2002). Thus, the current literature suggests the increased incidence of autoimmune disorders with age may result from a reduction in immune regulatory function.

In this study, we describe for the first time a decline in the number and immunoregulatory function of IL10-producing B cells with age, which is negatively correlated with the age-related increase in the circulating autoantibody rheumatoid factor, providing an important additional mechanism for the age-related increase in autoimmunity.

## Results

### Effect of age on the frequency of circulating CD19^+^CD24^hi^CD38^hi^ B cells

**Fig. 1:**
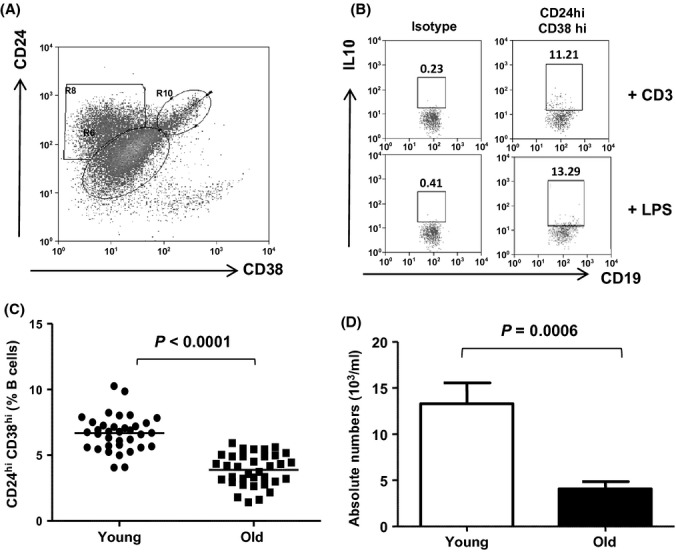
CD19^+^CD24^hi^CD38^hi^ B cells are the main producers of IL10 amongst B cells and their numbers decline with age. PBMCs were isolated from peripheral blood, stimulated using anti-CD3 antibody for 72 h and immunostained for expression of CD19, CD24, CD38 and intracellularly stained to measure IL10. (A) Representative flow cytometric plot showing the gating strategy used to identify regulatory B cells in the B-cell pool via expression of CD24 and CD38, (B) IL10 expression on B-cell subsets following T-cell-dependent stimulation via CD3 (upper panel) or T-cell independent stimulation via LPS (lower panel). (C) Scatter plot showing the percentages CD19^+^CD24^hi^CD38^hi^ B cells in peripheral blood of 35 healthy young donors and 35 healthy old donors without stimulation. The solid bar represents the mean value. (D) Bar chart showing the mean absolute number of CD19^+^CD24^hi^CD38^hi^ B cells in peripheral blood of healthy young and old donors.

Three major populations of circulating B cells have been reported in peripheral blood: transitional, circulating mature naïve and memory B cells discriminated on the basis of relative distribution of the markers CD24 and CD38. Functionally, immature transitional CD19^+^CD24^hi^CD38^hi^ B cells have been reported to comprise the highest percentage of IL10-producing B cells upon CD40 stimulation (Blair *et al*., 2010). Peripheral blood mononuclear cells (PBMC) isolated from young and older subjects were stimulated for 72 h with anti-CD3 antibody prior to immunostaining for the expression of CD19, CD24, CD38 and IL10 in order to identify B cells with regulatory properties (Blair *et al*., 2010). The gating strategy used to identify these cells is shown in Fig. 1A. Our findings are consistent with the previous literature that has reported that CD19^+^CD24^hi^CD38^hi^ cells are the main IL10-producing subset induced in a T-cell-dependent manner, (Fig. 1B, upper panel) compared with CD24^+ve^CD38^−ve^B (memory) and CD24^+ve^CD38^−ve^ (mature) B cells that produce little or no IL10 (Fig. S1 A). Similarly, we have also confirmed that CD19^+^CD24^hi^CD38^hi^ cells are the main IL10 producers in the B-cell pool upon LPS stimulation (Fig. 1B, lower panel), whereas CD24^+ve^CD38^−ve^B (memory) and CD24^+ve^CD38^+ve^ (mature) B cells produced very low levels of IL10 on LPS stimulation (Fig. S1 B). Tedder and colleagues have also reported that IL10 production was restricted to CD5^+^CD1d^hi^ B cells and referred to them as ‘B10’ cells showing that this subset expressed high levels of CD24 in mice (Yanaba *et al*., 2008a). IL10-producing B cells in humans are also CD5^+^CD1d^hi^CD24^hi^CD38^hi^ CD27^+^ (Iwata *et al*., 2011), but Mauri and colleagues have shown that the IgD^+ve^ CD27^−ve^ CD5^+^CD1d^hi^ B-cell subset was contained within the CD19^+^CD24^hi^CD38^hi^ population in humans (Blair *et al*., 2010). Despite this observation, we routinely assessed the CD19^+^CD5^+^CD1d^hi^ population of cells in our study and confirmed their presence in humans and that they responded to T-cell-mediated and TLR signalling by induction of IL10 (data not shown). As we found that the two populations behaved identically in each experiment, only the data for the CD19^+^CD24^hi^CD38^hi^ cells are presented.

Comparison of these subsets in healthy young and old subjects revealed that the percentage of both the CD19^+^CD24^hi^CD38^hi^ (Fig. 1C) and CD19^+^CD5^+^CD1d^hi^ B cells (Fig. S2 A) in the PBMC pool was significantly reduced in older adults. We also evaluated whether there was a numerical deficit in CD19^+^CD24^hi^CD38^hi^ and CD19^+^CD5^+^CD1d^hi^ B cells in old donors and found a significant reduction in absolute numbers of CD19^+^CD24^hi^CD38^hi^ B cells (Fig. 1D) and CD19^+^CD5^+^CD1d^hi^ B cells (Fig. S2 B) coinciding with the well-documented decrease in total B cell numbers with age (Cancro *et al*., 2009).

### Impaired IL10 production by B cells with age

**Fig. 2:**
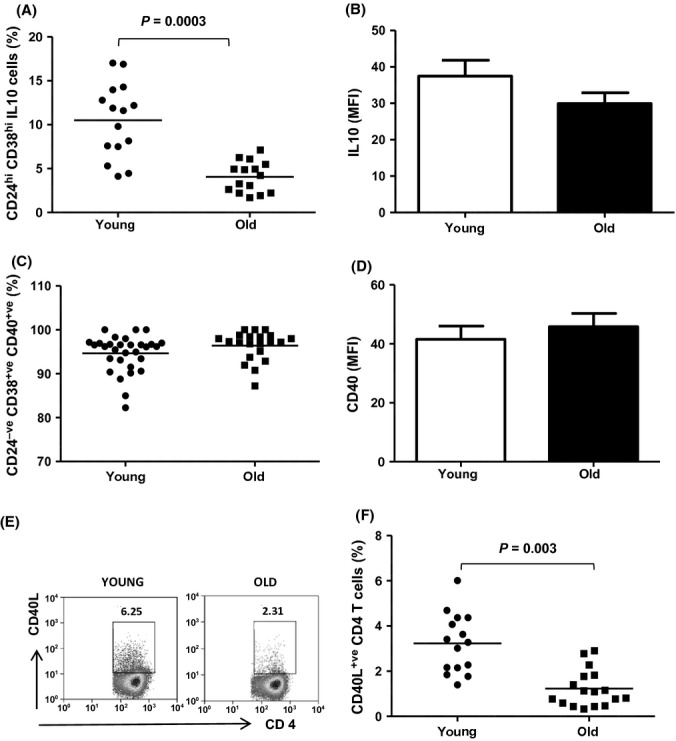
Impaired IL10 production by CD19^+^CD24^hi^CD38^hi^ B cells on CD3 stimulation with age is due to impaired T-cell helper activity. PBMCs isolated from 15 healthy young and older adults were stimulated via CD3 for 72 h and stained for surface expression of CD19, CD24, CD38 and intracellularly for IL10. (A) Scatter plots show the percentage of IL10 positive cells within the CD19^+^CD24^hi^CD38^hi^ B-cell subset in healthy young and old donors; (B) Bar charts showing the mean fluorescence intensity of IL10 staining within CD19^+^CD24^hi^CD38^hi^ B cells after 72 h; (C) PBMCs isolated from 30 healthy young and older adults were stained for CD19, CD24, CD38 and CD40. Scatter plot shows the percentage of CD40^+ve^CD24^hi^CD38^hi^ B cells in unstimulated peripheral blood of healthy young and aged donors; (D). Bar chart comparing the expression of CD40 (MFI) on CD19^+^CD24^hi^CD38^hi^ B cells without stimulation between young and old donors; (E) Representative flow cytometric plots showing the percentage of CD40L^+^ CD4T cells in healthy young and old donors after CD3 stimulation for 72 h; (F) Scatter plots showing the mean percentage of CD40L^+^ CD4T cells in PBMCs of 15 healthy young and older donors.

The hallmark of the immunosuppressive function of B cells is their ability to produce IL10 (Blair *et al*., 2009). To examine the effect of aging on immunosuppressive properties of B cells, we first determined the percentage of IL10 expressing CD19^+^CD24^hi^CD38^hi^ and CD19^+^CD5^+^CD1d^hi^ B cells following stimulation of T cells in the PBMC pool with anti-CD3 mAb for 72 h. We observed a significant decline in the percentage of IL10 expressing CD19^+^CD24^hi^CD38^hi^ (Fig. 2A) and CD19^+^CD5^+^CD1d^hi^ B cells (data not shown) in older individuals. We also compared the level of IL10 expressed by the individual CD19^+^CD24^hi^CD38^hi^ B cells, but did not observe any significant differences in the mean fluorescence intensity (MFI) values after immunostaining for IL10 expression by CD19^+^CD24^hi^CD38^hi^ cells (Fig. 2B). Previous studies in human CD19^+^CD24^hi^CD38^hi^ B cells have routinely included 6-h stimulation with PMA and ionomycin at the end of the 72-h anti-CD3 treatment (Blair *et al*., 2010). However, the inclusion of PMA and ionomycin treatment did not rescue the impaired induction of IL10 expressing CD19^+^CD24^hi^CD38^hi^ B cells in PBMC from old donors (data not shown). The effect of age is thus at the stage of induction of immature transitional CD19^+^CD24^hi^CD38^hi^ and CD19^+^CD5^+^CD1d^hi^ B10 B -cell differentiation into IL10-producing cells.

### Decline in CD4 T-cell helper activity with age

T cells activated through the TCR/CD3 pathway provide a costimulatory signal for inducing IL10 secretion by CD19^+^CD24^hi^CD38^hi^ B cells via upregulation of CD40 ligand (CD154). To investigate the possible cause of impaired IL10 production with aging, we examined the stability of CD40 expression on CD19^+^CD24^hi^CD38^hi^ B cells and CD40L expression on CD4 T cells with age. The proportion of CD40 positive B cells and the CD40 expression levels on the total B-cell pool remained stable with age (data not shown), as previously reported (Blaeser *et al*., 2008). Similarly, the percentage of CD40 expressing CD19^+^CD24^hi^CD38^hi^ B cells (Fig. 2C) and the CD40 expression levels on CD19^+^CD24^hi^CD38^hi^ cells (Fig. 2D) were comparable between young and aged individuals. In contrast, immunostaining for CD40L revealed a significant age-related decline in the percentage of basal CD4^+^ CD40L^+^ T cells (data not shown) and a significant reduction in CD3-induced CD40L expression on CD4 T cells in old donors (Fig. 2E and 2F). Although the level of CD40L induction is low, these figures are in line with a previous report that recorded 7.8% of T cells expressing CD40L after 72-h stimulation (Kosmaczewska *et al*., 2006). With age, CD4 T cells thus show a decline in their ability to provide an activating stimulus to B cells, which would contribute to impaired induction of IL10.

### Intrinsic defects in CD19^+^CD24^hi^CD38^hi^ B cells of older adults result in impaired IL10 production

**Fig. 3:**
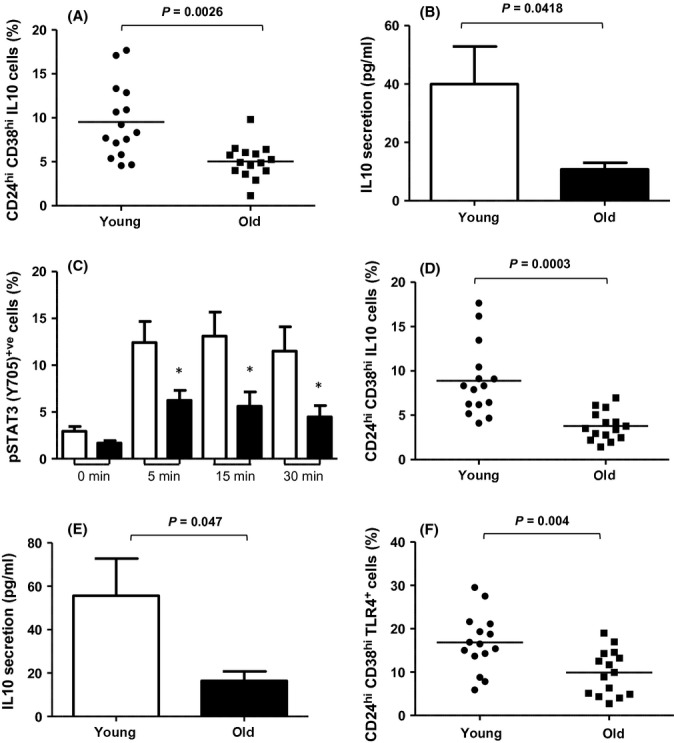
B cells from old donors display impaired IL10 secretion upon CD40 (T-cell dependent) and LPS (T-cell independent) stimulation. B cells isolated from 15 young, and older adults were stimulated with recombinant CD40L for 48 h and immunostained for surface expression of CD19, CD24, CD38 and intracellularly for IL10, and IL10 secretion was measured by ELISA in culture supernatants. (A) Scatter plot showing the mean percentage of IL10 positive cells within the CD19^+^CD24^hi^CD38^hi^ B-cell subset in young and old donor; (B) Bar graphs represent the mean IL10 concentrations (± SEM) in cell culture supernatants; (C) PBMCs from five young and old donors were stimulated with an agonistic anti-CD40 antibody and cells fixed and stained for phosphorylated STAT3 (pY705) at the time points shown. Data are the mean ± SD for the percentage of CD19^+^CD24^hi^CD38^hi^ B cells expressing pSTAT3 and * indicates *P* < 0.05; B cells isolated from 15 young and older adults were stimulated with LPS for 48 h and immunostained for surface expression of CD19, CD24, CD38 and intracellularly for IL10, and IL10 secretion was measured by ELISA in culture supernatants. (D) Scatter plot showing the mean percentage of IL10 positive cells within the CD19^+^CD24^hi^CD38^hi^ B-cell subset; (E) Bar graphs showing the mean IL10 concentration (± SEM) in cell culture supernatants; (F) PBMCs isolated from 15 young and older adults were stained with CD19, CD24, CD38 and TLR4. Scatter plot showing the percentage of TLR4^+ve^CD19^+^CD24^hi^CD38^hi^ B cells in peripheral blood without stimulation.

Next, we evaluated whether CD19^+^CD24^hi^CD38^hi^ and CD19^+^CD5^+^CD1d^hi^ B cells also exhibit cell-intrinsic defects in response to CD40 stimulation. Isolated B cells were stimulated for 48 h with recombinant CD40L, and a significant age-related decline in the induction of IL10 expression was observed in CD19^+^CD24^hi^CD38^hi^ (Fig. 3A) and CD19^+^CD5^+^CD1d^hi^ B cells (data not shown) in older individuals. Additionally, we also measured IL10 secretion by B cells after stimulation (48 h) with recombinant CD40L. Assessment of IL10 in culture supernatants by ELISA showed that IL10 secretion was significantly lower by cells from old donors (Fig. 3B). The impaired IL10 production upon direct stimulation with CD40L suggests that intrinsic defects in CD40 signalling in aged IL10-producing B cells may be a contributing factor towards impaired induction of immunoregulatory properties.

CD40 signalling results in the activation of multiple signalling cascades including signal transducers and activators of transcription factor 3 (STAT3)(Hanissian & Geha, 1997). CD40-mediated STAT3 activation is known to induce IL10 gene expression. Due to the low numbers of CD19^+^CD24^hi^CD38^hi^ cells in peripheral blood, we assessed STAT3 phosphorylation at tyrosine residue Y705 on a single-cell basis using phosphoflow cytometry. The percentage of CD19^+^CD24^hi^CD38^hi^ cells expressing phosphorylated STAT3 was significantly lower in cells from aged donors poststimulation with anti-CD40 mAb (Fig. 3C).

### Impaired IL10 production by B cells in older adults – T-cell independent stimulation

Toll-like receptor (TLR)-mediated signals can activate protective B-cell responses and provide a link between microbial recognition and suppression of autoimmune diseases. We therefore also examined the effect of TLR4 stimulation via lipopolysaccharide (LPS) for 48 h on CD19^+^CD24^hi^CD38^hi^ and CD19^+^CD5^+^CD1d^hi^ B cells in young and older adults. We saw a significant decline in induction of IL10 expression by CD19^+^CD24^hi^CD38^hi^ (Fig. 3D) and CD19^+^CD5^+^CD1d^hi^ B cells (data not shown) on LPS stimulation in older individuals. We also examined IL10 secretion by B cells and found significantly lower IL10 levels in the culture supernatant from old donor B cells (Fig. 3E). To investigate the basis of the impaired IL10 induction on LPS stimulation by CD19^+^CD24^hi^CD38^hi^ B cells, we examined TLR4 expression on B cells from young and old donors. We observed a significant decline in the percentage of total TLR4-expressing B cells (data not shown) and specifically in CD19^+^CD24^hi^CD38^hi^ B cells (Fig. 3F) in older adults. However, we did not detect any significant differences in protein expression on a per cell basis for TLR4 on CD19^+^CD24^hi^CD38^hi^ cells with age (data not shown).

### CD80 and CD86 expression on CD19^+^CD24^hi^CD38^hi^ B cells with age

**Fig. 4:**
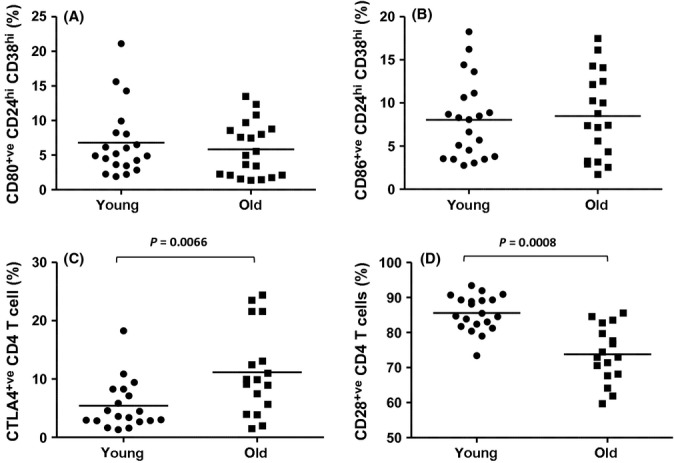
Effect of age on CD80 and CD86 expression on CD19^+^CD24^hi^CD38^hi^ B cells and CTLA4 and CD28 expression on CD4 T cells. PBMCs isolated from 20 young and older adults were stained with CD19, CD24, CD38 and CD80 or CD86. Scatter plots showing the effect of age on the percentage of (A) CD80^+ve^CD24^hi^CD38^hi^ B cells or (B) CD86^+ve^CD24^hi^CD38^hi^ B cells in peripheral blood without stimulation; Scatter plots showing the percentage of (C) CTLA4^+^ CD4T cells and (D) CD28^+^ CD4T cells in PBMCs of 20 young and older donors.

Previous studies have reported that the suppressive activity of B cells is not exclusively IL10 dependent (Mizoguchi & Bhan, 2006;. Engagement of CD80/CD86 with CD28 and CTLA4 expressed on T cells has been proposed as an additional mechanism of suppression by regulatory B cells (Blair *et al*., 2010). We found that young and aged donor B cells did not differ in the percentage of cells expressing costimulatory molecules CD80 and CD86 (data not shown) or in the levels of expression of costimulatory molecules (data not shown). Similarly, we found no differences between the percentage of CD19^+^CD24^hi^CD38^hi^ B cells expressing costimulatory molecules CD80 (Fig. 4A) and CD86 (Fig. 4B) or in the levels of expression of costimulatory molecules in these cells (data not shown).

Additionally, we examined the expression of CTLA4 and CD28 on CD4 T cells with age and observed a significant increase in percentage of CTLA4 expressing CD4 T cells (Fig. 4C), even though the expression levels remained unaltered (data not shown). These findings are consistent with previous reports of CTLA4 upregulation with age (Leng *et al*., 2002). We also observed a significant decline in the percentage of CD28 expressing CD4 T cells with age (Fig. 4D) that is consistent with previous findings (Vallejo, 2005). However, we saw no differences in the expression levels of CD28 on CD4 T cells between young and aged donors (data not shown).

### CD19^+^CD24^hi^CD38^hi^ B cells and autoimmunity

**Fig. 5:**
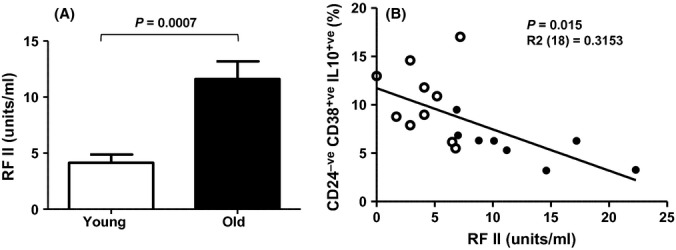
Autoantibody levels increase with age and correlate with reduced IL10 production by CD19^+^CD24^hi^CD38^hi^ B cells. (A) Serum levels of rheumatoid factor (RF) were measured in 10 healthy old and 10 healthy young donors. Data are mean ± SD. (B) Serum RF values were plotted against ability of CD19^+^CD24^hi^CD38^hi^ B cell to produce IL10 upon CD3 stimulation of PBMCs in young (open circles) and old (closed circles) donors.

The incidence of autoimmunity increases with age (Lindstrom & Robinson, 2010). For example, approximately 25% of older adults have detectable levels of low-affinity autoantibodies in their blood, including rheumatoid factors (RFs), which are present in only 5% of young healthy individuals (Moulias *et al*., 1984). Here, we have shown a numerical and functional impairment in the subset of IL10-producing B cells with age, and we thus questioned whether this impairment may contribute towards increased risk of autoimmunity with age. On testing serum RF levels of healthy young and aged individuals, we observed a significant increase in serum RF levels in older adults compared with young adults (Fig. 5A) and a significant negative correlation between RF antibody titre levels and IL10 production by CD19^+^CD24^hi^CD38^hi^ B cells (*P* = 0.015; Fig. 5B). These results suggest that age -associated impairment in the ability of CD19^+^CD24^hi^CD38^hi^ B cells to produce IL10 with age may be linked with the elevated risk of autoimmunity with age.

## Discussion

Advancing age is associated with remodelling of the immune system that predisposes elders to the development of autoimmune disorders such as rheumatoid Arthritis (Lindstrom & Robinson, 2010). These changes include reduced levels of circulating IL10 with raised levels of pro-inflammatory cytokines, termed ‘inflammaging’ (Franceschi, 2007) and Tregs with reduced immune suppression activity in older adults (Dejaco *et al*., 2006). Aging is also accompanied by compromised peripheral tolerance, represented by increased levels of autoantibodies in the circulation, suggesting a physiological impact of reduced T regulatory cell function. However, studies over the past decade have challenged the paradigm of Treg cells as the sole cell type involved in regulation of immune responses and B cells with immunosuppressive properties have also been identified in humans (Blair *et al*., 2010; Iwata *et al*., 2011). Furthermore, the ability of B cells to secrete IL10 has been shown to be impaired in autoimmune diseases, including SLE (Blair *et al*., 2010) and multiple sclerosis (Duddy *et al*., 2007), and treatment of multiple sclerosis patients with mitoxantrone, an approved chemotherapy for aggressive MS, results in increased B-cell IL10 production and lower production of the pro-inflammatory cytokines TNF and lymphotoxin (Neuhaus *et al*., 2005).

Here, we report a profound effect of aging on the frequency and immunosuppressive function of transitional CD19^+^CD24^hi^CD38^hi^ and CD19^+^CD5^+^CD1d^hi^ B10 B cells, resulting from both a lack of T cell help due to reduced upregulation of CD40L upon activation and reduced signalling through CD40 on the B cell. Previous studies have reported impairments in CD4^+^ T-cell helper activity with age, namely a reduction in CD40L upregulation by CD4 T cells on stimulation in mice (Eaton *et al*., 2004) and humans (Fernandez-Gutierrez *et al*., 1999). Reduced CD40L expression on CD4 T cells with age has also been associated with increased levels of dual-specific phosphatase 4 (DUSP4) in CD4 T cells from old donors (Yu *et al*., 2012). In addition, impaired coalescence of lipid rafts in T cells of older adults following stimulation through CD3 has been reported (Larbi *et al*., 2004) and might also play a role in reducing T cell help for IL10-producing B cells. That aging is accompanied by intrinsic defects in B cells themselves has also been reported in mice. Adoptive transfer studies in severe combined immunodeficient (SCID) mice revealed that antibodies produced by B cells from young mice, when paired with T cells from aged donors, had a reduced rate of somatic mutation and VH gene usage. However, when aged B cells were transferred with young T cells, somatic mutation still failed to accumulate normally (Yang *et al*., 1996). Another study highlighting intrinsic defects in B cells with age reported a decrease in transcription factor E47 expression on anti-CD40 stimulation, resulting in decreased class switching in aged murine B cells (Frasca *et al*., 2003). Our data show that age-related intrinsic B-cell defects extend to CD19^+^CD24^hi^CD38^hi^ and CD19^+^CD5^+^CD1d^hi^ cells in humans as we have shown impaired IL10 induction by these immunosuppressive B cells upon direct stimulation with recombinant CD40L and reduced phosphorylation of STAT3. These data are consistent with a previous report of impaired STAT3 signalling with age in human lymphocytes (Fulop *et al*., 2006). Additionally, CD19^+^CD24^hi^CD38^hi^ B cells from SLE patients have been shown to have impaired IL10 production upon CD40 activation, and this was correlated with lower levels of STAT3 (Blair *et al*., 2010).

A link between reduced B-cell immunoregulatory function and age-related autoimmunity is supported by our data showing a negative correlation between CD19^+^CD24^hi^CD38^hi^ B-cell IL10 production and serum Rf levels in our subjects. Previous findings have reported an exacerbated disease phenotype and enhanced mortality in IL10 gene-deficient lupus-prone MLR mice, and administration of rIL-10 reduced autoantibody production, highlighting the importance of IL10 in down modulating autoimmunity (Yin *et al*., 2002). Others have shown that mice lacking IL10-producing B cells develop an exacerbated antigen-induced arthritis, which is also associated with reduced numbers of T_regs_ and increased Th1 and Th17 cells (Carter *et al*., 2011). Additionally, B7 expression by B cells is also required for their regulatory function, mediating peak expression of IL10 and Foxp3 (Salomon & Bluestone, 2001). However, we did not observe any difference in levels of costimulatory molecules on CD19^+^CD24^hi^CD38^hi^ with age. IL10-producing B cells also promote induction of regulatory T-cell function over inflammatory T-cell function. The reduced functioning of CD19^+^CD24^hi^CD38^hi^ and CD19^+^CD5^+^CD1d^hi^ B cells seen here might therefore contribute not only to loss of T_reg_ function with age (Dejaco *et al*., 2006), helping to promote autoimmunity, but also to the age-related increase in systemic inflammation. IL10 modulates the inflammatory function of monocytes, and the raised level of basal monocyte cytokine output with age has been reported in both aged mice (Arranz *et al*., 2010) and humans (Franceschi, 2007), termed inflammaging. Reduced regulatory B-cell function could therefore also contribute to this age-related phenomenon.

The literature also contains reports of age-associated defects in TLR-induced cytokine production in monocytes, macrophages and dendritic cells (Panda *et al*., 2010), including reduced TLR4 expression and function on macrophages and dendritic cells with age (Renshaw *et al*., 2002). We have shown a significant decline in IL10 production by B cells in old donors upon LPS stimulation and a reduction in TLR4 expression on aged CD19^+^CD24^hi^CD38^hi^ and CD19^+^CD5^+^CD1d^hi^ B cells that may contribute to reduced responsiveness to LPS, which is also seen in monocytes and DCs with age (Qian *et al*., 2012). TLR-signalling pathways in IL10-producing B cells were not investigated here, but may also contribute to altered IL10 secretion upon LPS stimulation in older adults. MyD88 is required for optimal IL10 production and secretion following LPS stimulation (Yanaba *et al*., 2009).

Interestingly, a novel B-cell subset has been characterized recently by the Marrack group and suggested to play a role in age-related autoimmunity: the CD11c^+^ B cell increases in frequency in aged female mice leading to the name ‘age-associated B cell (ABC)’. These cells are also detected in young lupus-prone mice and elderly women with autoimmune disease (Rubtsov *et al*., 2011). Although we have not determined the presence of ABCs in our study, it is interesting to postulate that their age-related increase is accompanied by a decline in IL10-producing B cells, representing a remodelling of the B-cell pool towards autoimmune-enhancing cells.

In summary, our findings demonstrate that the frequency of CD19^+^CD24^hi^CD38^hi^ and CD19^+^CD5^+^CD1d^hi^ B cells and their ability to produce IL10 after *ex vivo* maturation/stimulation declines with age and that this correlates with an age-related increase in serum autoantibody (RF) in healthy older adults. Reduced induction of IL10 secretory function was due to both compromised intrinsic B-cell signalling through CD40 and reduced expression of CD40L on CD4 T cells.

## Experimental procedures

### Subjects

In total, 56 young (mean age 26.81, range 20–36 years) and 65 old (mean age 70.12, range 60–84 years) subjects participated in this study. A venous blood sample was taken from each subject between the hours of 09:00–11:00 am after obtaining written informed consent. At the time of blood sampling, none of the subjects had an acute infection or were taking any medication known to alter immune function (such as steroids or statins) and none had any chronic illness. The study has been approved by the North Staffordshire Research Ethics Committee.

#### Human cell isolation and culture

Peripheral blood mononuclear cells (PBMC's): PBMCs were isolated from peripheral blood by density centrifugation using Ficoll-Paque™ PLUS (GE Healthcare, Uppsala, Sweden). Isolated PBMC's were resuspended in RPMI medium containing 2 mm L-glutamine, 100 U ml penicillin, 100 μg ml streptomycin (Sigma-Aldrich, Dorset, UK) supplemented with 10% heat-inactivated fetal calf serum (Sera Laboratories International, Sussex, UK) at a concentration of 1 × 10^6^ ml^−1^ for functional and phenotypic analysis.

#### Immunostaining for phenotypic analysis of B cells and T cells

Isolated PBMCs were stained with a combination of fluorochrome-conjugated antibodies including: CD19-PE (clone HIB19; eBiosciences, Hatfield, UK), CD24-FITC (clone eBioSN3; eBiosciences), CD38-PEcy7 (clone HIT2; eBiosciences), CD19-FITC (clone H1B19; eBiosciences), CD5-PEcy7 (clone UCHT2; eBiosciences), CD1d-PE (clone 51.1; eBiosciences), CD40-APC(clone 5C3; eBiosciences), TLR4 (CD284)-APC (clone HTA125; eBiosciences), CD80-APC (clone 2D10; Bio legend, London, UK), CD86-APC (clone IT2.2; Bio legend), CD3-FITC (Dako, cloneUCHTI), CD4-PE (clone RPA-T4; eBiosciences), CD154-APC (clone 24–31; eBiosciences), CTLA4- APC (clone L3D10; Bio legend) and CD28-APC (clone CD28.2; BD Biosciences) Appropriate isotype controls were used for setting gates. Following incubation, cells were washed and resuspended in PBS for flow cytometric analysis using a Cyan TM ADP flow cytometer (Dako). Mean data are expressed as MFI and as a natural number rather than on the log scale.

#### B-cell functional assays – CD3 stimulation

Prior to set-up of stimulation, wells of a 96-well microtitre plates with round bottom wells (Sarstedt Ltd, Leicester, UK) were coated with anti-CD3 mAb (BD Biosciences) at a concentration of 0.5 μg ml for 1 h at 37° C. Isolated PBMCs were plated at 25 × 104 cells/well for 72 h at 37° C in a humidified atmosphere of 5% CO_2_. Brefeldin A (10μg ml^−1^; Sigma-Aldrich) with PMA (50 ng ml^−1^; Sigma-Aldrich) and ionomycin (500 ng ml^−1^; Sigma-Aldrich) were added during the last 6 h of the incubation. Postculture PBMCs were stained with extracellular surface expression markers CD3-FITC (Dako, cloneUCHTI), CD4-PE (clone RPA-T4; eBiosciences) and CD154-APC (clone31–24; eBiosciences) to measure CD40L (CD154) expression on CD3-stimulated CD4 T cells.

#### B-cell isolation and stimulation – CD40L and LPS

B cells were isolated from PBMCs by negative selection using MACS® technology (Human B cell isolation kit; Miltenyi Biotech UK, Gladbach, Germany) following the manufacturer's instruction. Isolated B cells were resuspended in complete medium at 1 × 10^6^ ml^−1^. Purity of isolated B cells was routinely assessed and was ≥ 92%. B cells were stimulated with either recombinant human CD40L (1 μg ml^−1^; Peprotech, Hull, UK) or LPS isolated from *Escherichia coli* serotype 0111:B4 (1 μg ml^−1^; Sigma- Aldrich) for 48 h. Brefeldin A (10 μg ml^−1^; Sigma-Aldrich) was added during the last 6 h of the stimulation. At the end of incubation, B cells were washed with PBS.

#### Flow cytometric detection of intracellular IL10

After culturing cells with stimulus, they were washed and stained for a combination of extracellular surface markers to identify IL10-producing transitional B cells (CD19 PE, CD24 FITC and CD38 PEcy7) and B10 B cells (CD19 FITC, CD5 PEcy7, CD1d PE). Poststaining cells were fixed (Reagent A; Fix and Perm kit, Invitrogen Ltd, Paisley, Scotland) and permeabilized (Reagent B; Fix and Perm kit, Invitrogen). Permeabilized cells were stained with anti-human Alexa fluor 647 IL10 antibody (clone JES3-9D7) for intracellular staining. Appropriate APC-conjugated isotype controls were used for gate setting for measuring cytokine expression.

#### Quantification of IL10 levels in supernatant

Isolated B cells (1 × 10^6^ cells) were stimulated with either recombinant human CD40L (1 μg ml^−1^; Peprotech, Hull, UK) or LPS isolated from *Escherichia coli* serotype 0111:B4 (1 μg ml^−1^; Sigma-Aldrich) for 48 h (as described above). Poststimulation, the supernatant was collected and IL10 levels were quantified using a Human IL-10 ELISA (Abcam, UK) as per manufacturer's instructions.

#### Measuring phosphorylation of STAT3

PBMCs were rested for 1 h in medium after which cells were stimulated with purified agonistic anti-human CD40 mAb (eBiosciences) at 0.5 μg 10^6^ per cells for up to 30 min in the dark at 4° C. Poststimulation cells were washed and stained for a combination of extracellular surface markers to identify transitional B cells (CD19-PE, CD24-FITC and CD38-PEcy7) and were then fixed immediately to maintain phosphorylation state by adding 100 μl of 1.6% paraformaldehyde for 30 min at room temperature in the dark. Postfixation cells were washed and permeabilized using methanol and stained with anti-STAT3 (pY705) phosphospecific antibody (BD Biosciences, Oxford, UK) and appropriate isotype controls for 30 min in the dark at 4° C. Cells were analysed using three and four channel flow cytometry on a Cyan™ ADP (Dako). Data analysis was carried out using Summit v4.3x software (Dako).

#### Statistical analysis

Statistical analysis was performed using GraphPad Prism® (Graph Pad, La Jolia, USA) software. Data distribution was checked for normality using the Kolmogorov-Smirnov test. For normally distributed data, a Student's *t*-test analysis was performed to assess differences between two conditions. *P*-values of < 0.05 were considered significant.

## Supporting Information

**Fig. S1** PBMCs were isolated from peripheral blood, stimulated using (A) anti-CD3 antibody or (B) LPS for 72 h and immunostained for expression of CD19, CD24, CD38 and intracellularly stained to measure IL10.

**Fig. S2** PBMCs were isolated from peripheral blood, stimulated using anti-CD3 antibody for 72 h and immunostained for expression of CD19, CD1d, CD5 and intracellularly stained to measure IL10.
