# Supplementary figures and images for: An age-related numerical and functional deficit in CD19+CD24hiCD38hi B cells is associated with an increase in systemic autoimmunity
Source: Aging Cell. 2013 Jul 19;12(5):873–81. doi: 10.1111/acel.12114 (PMC3814412; doi:10.1111/acel.12114)

# Supplementary figures

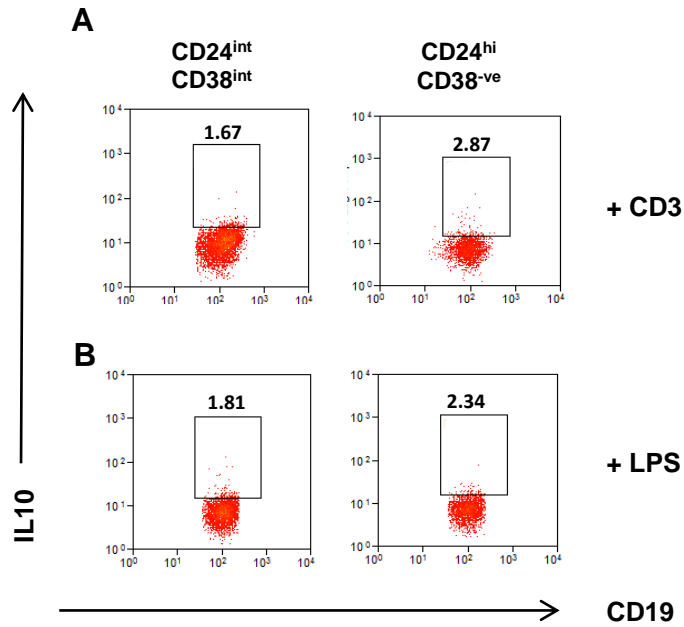

Figure 1

## Supplementary figures

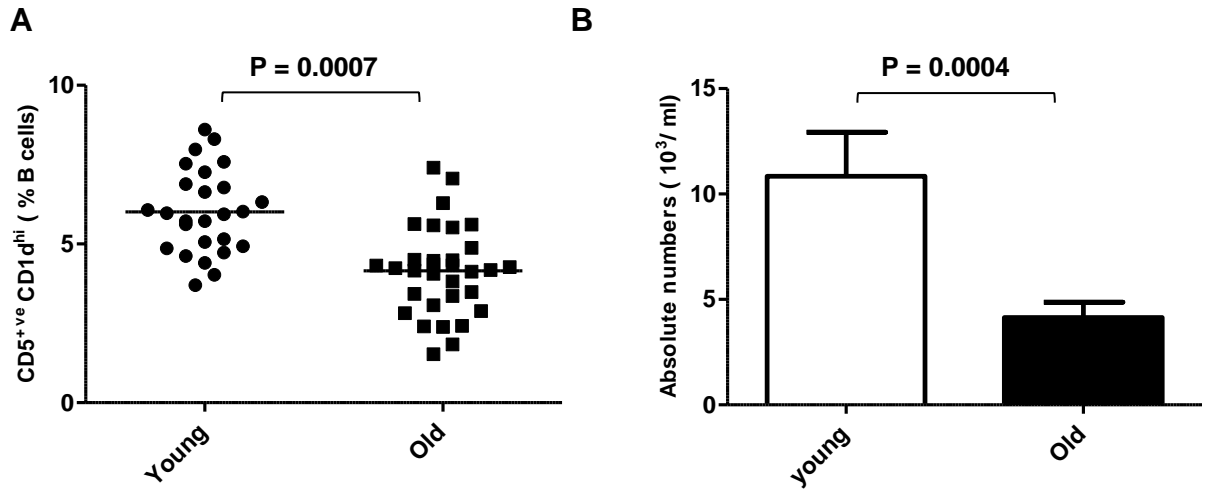

Figure 2

Supplement: Supplementary file 1 [file acel0012-0873-SD1.pdf]

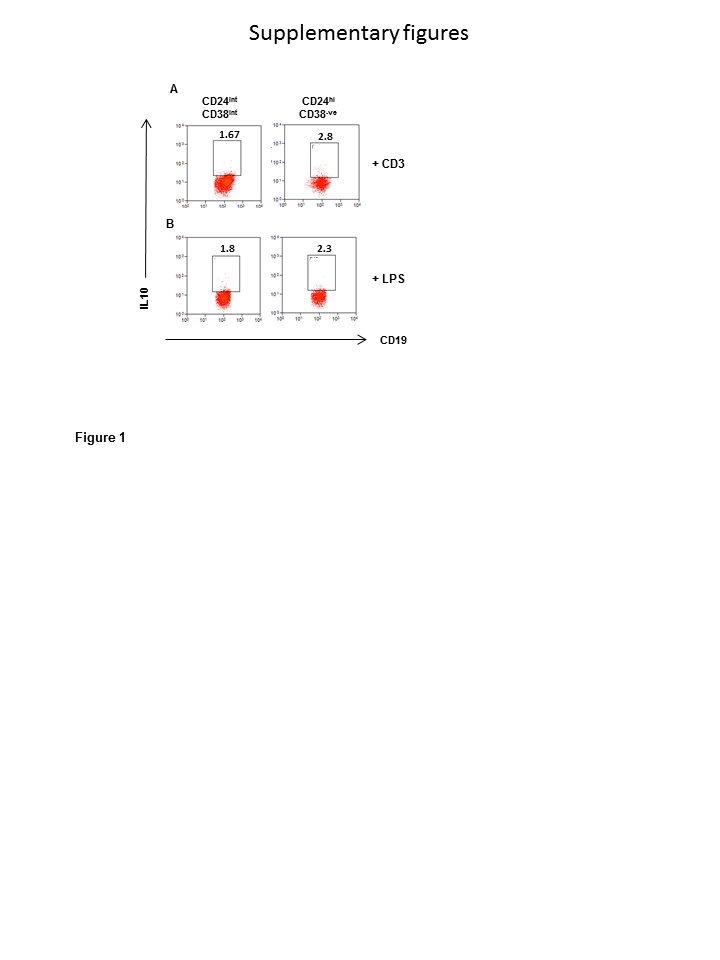

Supplement: Supplementary file 2 [file acel0012-0873-SD2.tif]

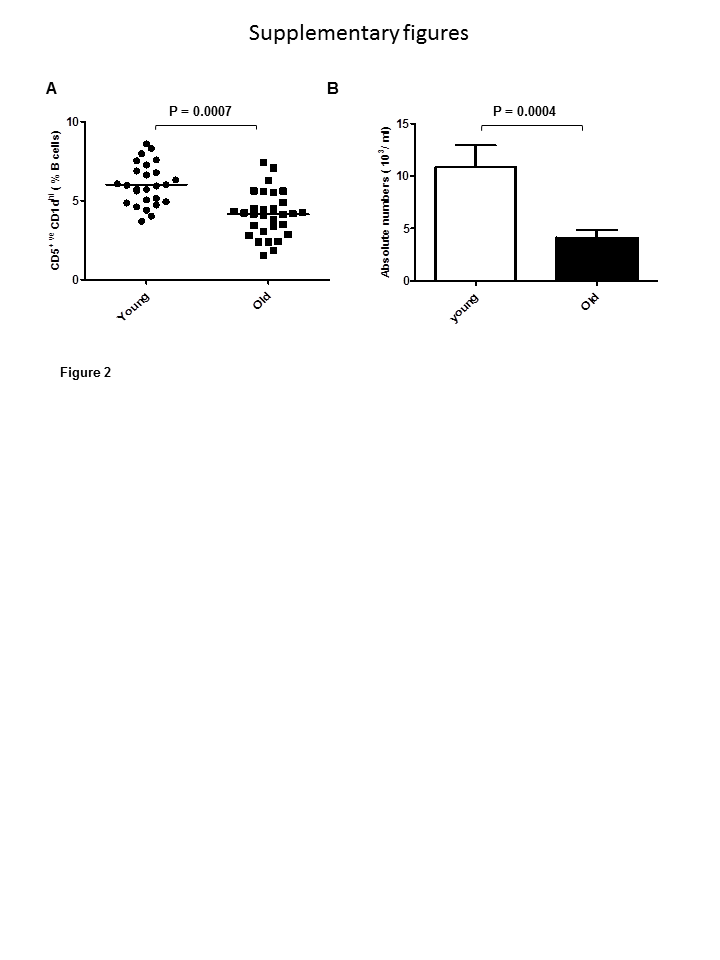

Supplement: Supplementary file 3 [file acel0012-0873-SD3.tif]
